# *Pinus densiflora* leaf essential oil induces apoptosis via ROS generation and activation of caspases in YD-8 human oral cancer cells

**DOI:** 10.3892/ijo.2011.1263

**Published:** 2011-11-11

**Authors:** JEONG-RANG JO, JU SUNG PARK, YU-KYOUNG PARK, YOUNG ZOO CHAE, GYU-HEE LEE, GY-YOUNG PARK, BYEONG-CHURL JANG

**Affiliations:** 1 Department of Medical Genetic Engineering, Keimyung University School of Medicine, Daegu 704-701; 2 Seoul Metropolitan Government Research Institute of Public Health and Environment, Woosong University, Daejeon 300-718; 3 Department of Food Science and Technology, Woosong University, Daejeon 300-718; 4 Department of Rehabilitation and Medicine, College of Medicine, Catholic University of Daegu, Daegu 705-717, Republic of Korea

**Keywords:** pine leaf essential oil, apoptosis, ROS, caspase-9, Bcl-2, YD-8

## Abstract

The leaf of *Pinus (P.) densiflora*, a pine tree widely distributed in Asian countries, has been used as a traditional medicine. In the present study, we investigated the anticancer activity of essential oil, extracted by steam distillation, from the leaf of *P. densiflora* in YD-8 human oral squamous cell carcinoma (OSCC) cells. Treatment of YD-8 cells with *P. densiflora* leaf essential oil (PLEO) at 60 μg/ml for 8 h strongly inhibited proliferation and survival and induced apoptosis. Notably, treatment with PLEO led to generation of ROS, activation of caspase-9, PARP cleavage, down-regulation of Bcl-2, and phosphorylation of ERK-1/2 and JNK-1/2 in YD-8 cells. Treatment with PLEO, however, did not affect the expression of Bax, XIAP and GRP78. Importantly, pharmacological inhibition studies demonstrated that treatment with vitamin E (an anti-oxidant) or z-VAD-fmk (a pan-caspase inhibitor), but not with PD98059 (an ERK-1/2 inhibitor) or SP600125 (a JNK-1/2 inhibitor), strongly suppressed PLEO-induced apoptosis in YD-8 cells and reduction of their survival. Vitamin E treatment further blocked activation of caspase-9 and Bcl-2 down-regulation induced by PLEO. Thus, these results demonstrate firstly that PLEO has anti-proliferative, anti-survival and pro-apoptotic effects on YD-8 cells and the effects are largely due to the ROS-dependent activation of caspases.

## Introduction

Oral squamous cell carcinoma (OSCC) is one of the most common malignant cancers of the oral cavity. OSCC, being one of the most disfiguring type of cancer, often causes a lack of quality of life in patients, including a loss of general cognitive, social, emotional or physical functions and prompting isolation (1). It is suggested that the primary treatment of OSCC is surgical removal, but radiation and/or chemotherapy are alternative treatment regimens against OSCC (2,3). Characteristically, OSCC cells, at the late stage of malignancies, are very resistant to cancer therapy-mediated apoptosis. Thus, research towards not only understanding and overcoming resistance mechanism but also identification of new drugs or substances with better preventive and/or therapeutic efficacy against OSCC is needed.

Many therapeutic and chemopreventive agents eliminate cancer cells by inducing apoptosis, a form of cell death. Apoptosis is well characterized with distinct morphological changes, including plasma membrane blebbing, depolarization of mitochondria and DNA fragmentation (4). Induction of apoptosis is mediated by a variety of cellular proteins and/or factors. Caspases are the essential proteases for the execution of cell death by apoptotic stimuli (5). The members of B cell lymphoma (Bcl-2) family or inhibitor of apoptosis (IAP) family are anti-apoptotic proteins, and their expressions are critical for cell survival and/or death (6–8). Oxidative stress is another key factor linked to induction of apoptosis (9,10). A role of endoplasmic reticulum (ER) stress in induction of apoptosis also has been reported (11,12). There is also substantial evidence to indicate involvement of activities of some intracellular signaling proteins, such as the family of mitogen-activated protein kinase (MAPK), in induction of apoptosis (13,14).

Essential oil is a volatile, natural and complex compound present in a variety of aromatic plants and mostly extracted by steam or hydro-distillation from the plants. In nature, plant-derived essential oil, due to its anti-bacterial, anti-viral, anti-fungal and/or insecticidal activities, play a role in the protection of the plants (15). It is now well accepted that some plants-derived essential oils are commercially important for food, sanitary, cosmetic and perfume industries (15–18), while others have potential to be developed as medicinal purposes, such as anticancer drugs. For example, it is shown that essential oil from *Myrica gale L.*, a native plant from Canada used in traditional medicine, has strong cytotoxic effects on human lung (A549) and colon (DLD-1) cancer cell lines (19) and that essential oil from *Ocimum basilicum L.* and *Psidium guajava L.*, Thai medicinal plants, inhibits proliferation of murine leukemia (P388) and human mouth epidermal carcinoma (KB) cell lines, respectively (20). Moreover, a recent study demonstrates that essential oil from pine needle inhibits growth and induces apoptosis in human liver carcinoma cells by down-regulation of Bcl-2 expression and telomerase activity (21).

The leaf of *Pinus (P.) densiflora*, a pine tree widely distributed in Asian mountains, has been used as a traditional medicine (22). Little is known about the relationship between pine leaf-derived essential oil and oral malignancies. In this study, we evaluated the anti-proliferative, anti-survival and/or pro-apoptotic effects of essential oil extracted by steam distillation from the leaf of *P. densiflora* on human OSCC cells and determined the possible molecular and cellular mechanisms.

## Materials and methods

### Materials

Materials were purchased as follows: RPMI-1640 medium, fetal bovine serum (FBS) and penicillin-streptomycin were from WelGene (Daegu, Korea). Antibody of procaspase-9 was from Enzo Life Science (Farmingdale, NY, USA). Antibody of XIAP was from R&D Systems (Minneapolis, MN, USA). Antibodies of Bcl-2, Bax and glucose-regulated protein 78 (GRP78) were from Santa Cruz Biotechnology (Santa Cruz, CA, USA). Antibodies against phosphorylated forms of ERK-1/2 (p-ERK-1/2), total forms (both phosphorylated and non-phosphorylated) of ERK-1/2 (T-ERK-1/2), p-JNK-1/2 and T-JNK-1/2 were from Cell Signaling Technology (Danvers, MA, USA). Antibodies against poly (ADP-ribose) polymerase (PARP) were from Roche Diagnostics (Mannheim, Germany). Antibodies against goat anti-rabbit IgG-HRP and goat anti-mouse IgG-HRP were from Santa Cruz Biotechnology. Enzyme-linked chemiluminescence (ECL) Western detection reagents were from Thermo Scientific (Waltham, MA, USA). 3-(4,5-Dimethylthiazol-2-yl)-5-(3-carboxymethoxyphenyl)-2-(4-sulfophenyl)-2H-tetrazolium (MTS) reagent was from Promega (Madison, WI, USA). N-benzyloxycarbonyl-Val-Ala-Asp-fluoromethylketone (z-VAD-fmk) and proteinase inhibitor cocktail (x100) were from Calbiochem (Madison, WI, USA). Bradford reagent was from Bio-Rad (Hercules, CA, USA). Plasticware: 6-, 24- and 96-well plates and 60-mm cell culture dish was from SPL Life Sciences (Gyeonggi-do, Korea). Other reagents, including actin antibody, was from Sigma (St. Louis, MO, USA).

### Cell culture

Three human OSCC cell lines YD-8, YD-10B and YD-38 were purchased from Korean Cell Line Bank (Seoul, Korea). They all were grown at 37°C in a humidified condition of 95% air and 5% CO_2_ in RPMI-1640 supplemented with 10% heat-inactivated FBS, 100 units/ml penicillin and 100 μg/ml streptomycin.

### Cell proliferation assay

YD-8 cells (0.4×10^4^/100 μl/well) were seeded into 96-well plates overnight. Cells were then treated without or with different concentrations of PLEO for 8 h and incubated with MTS (20 μl/well) for 1.5 h at 37°C. The absorbance was measured at 595 nm using a microplate reader.

### Cell count analysis

Briefly, YD-8, YD-10B or YD-38 cells were seeded in 24-well plates (1×10^5^/500 μl/well) overnight. Respective cells were then treated without or with PLEO for 8 h. The number of surviving cells, which cannot be stained with trypan blue dye, was counted under microscope. Approximately, <100 cells were counted for the analysis.

### Measurement of DNA fragmentation

YD-8 cells were seeded in 6-well plates at a density of 0.5×10^6^ cells per well in 2 ml volume the day before PLEO treatment. Cells were incubated without or with different concentrations of PLEO for 8 h. Control or PLEO-treated cells were then harvested, washed and lysed in a buffer [50 mM Tris (pH 8.0), 0.5% sarkosyl, 0.5 mg/ml proteinase K and 1 mM EDTA] at 55°C for 3 h, followed addition of RNase A (0.5 μg/ml) and further incubation at 55°C for 18 h. The lysates were centrifuged at 10,000 × g for 20 min. The genomic DNA in the supernatant was extracted with equal volume of neutral phenol-chloroform-isoamyl alcohol mixture (25:24:1) and analyzed by electrophoresis on 1.7% agarose gel. The DNA was visualized and photographed under UV illumination after staining with ethidium bromide (0.1 μg/ml).

### Preparation of whole cell lysates

To see the effect of PLEO on total expression levels of cellular proteins, including procaspase-9, PARP, Bcl-2, Bax, XIAP, GRP78 and actin, YD-8 cells (0.5×10^6^/2 ml/well) were seeded in 6-well plates the day before PLEO treatment. Cells were treated without or with different concentrations of PLEO for 2, 4 or 8 h. Control or PLEO-treated cells were then washed twice with PBS and exposed to cell lysis buffer [50 mM Tris-Cl (pH 7.4), 150 mM NaCl, 0.1% sodium dodecyl sulfate, 0.25% sodium deoxycholate, 1% Triton X-100, 1% Nonidet P-40, 1 mM EDTA, 1 mM EGTA, proteinase inhibitor cocktail (x1)]. The cell lysates were collected in a 1.5-ml tube and centrifuged for 20 min at 4°C at 12,000 rpm. The supernatant was saved and protein concentrations were determined with Bradford reagent.

### Western blot analysis

Proteins (50 μg) were separated by SDS-PAGE (10%) and transferred onto nitrocellulose membranes (Millipore). The membranes were washed with TBS (10 mM Tris, 150 mM NaCl) supplemented with 0.05% (vol/vol) Tween-20 (TBST) followed by blocking with TBST containing 5% (wt/vol) non-fat dried milk. The membranes were incubated overnight with antibodies specific for procaspase-9 (1:2,000), PARP (1:2,000), Bcl-2 (1:1,000), Bax (1:2,000), XIAP (1:1,000), GRP78 (1:1,000), p-ERK-1/2 (1:1,000), T-ERK-1/2 (1:2,000), p-JNK-1/2 (1:1,000), T-JNK-1/2 (1:1,000) or actin (1:5,000) at 4°C. The membranes were then exposed to secondary antibodies coupled to horseradish peroxidase for 2 h at room temperature. The membranes were washed three times with TBST at room temperature. Immunoreactivities were detected by ECL reagents. Equal protein loading was assessed by the expression level of actin protein.

### Measurement of intracellular ROS

The generation of ROS was measured by a flow cytometry analysis using 2′,7′-dichlorfluorescein-diacetate (DCFH-DA) as a substrate. Briefly, YD-8 cells were grown in 60-mm cell culture dish at a density of 0.8×10^6^ cells in 2 ml volume overnight. YD-8 cells were loaded with DCFH-DA to a final concentration of 20 μM for 20 min and then treated without or with PLEO (60 μg/ml) for 10 min to 8 h. YD-8 cells were harvested, washed twice with PBS and suspended in PBS. The ROS generation was measured by the DCF fluorescence intensity (FL-1, 530 nm) from 10,000 cells with a FACS Caliber flow cytometer (Becton-Dickinson).

### Statistical analysis

MTS or cell count analysis was done in triplicates and repeated three times. Data are expressed as mean ± standard error (SE). The significance of difference was determined by One-Way ANOVA. All significance testing was based on upon a P-value of <0.05.

## Results

### Treatment with P. densiflora leaf essential oil (PLEO) inhibits proliferation and survival and induces apoptosis in YD-8 cells

**Figure 1:**
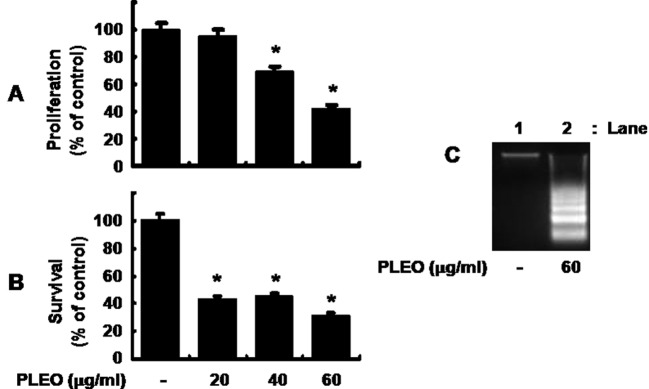
Effects of *P. densiflora* leaf essential oil (PLEO) on proliferation, survival and apoptosis in YD-8 cells. (A) YD-8 cells were treated without or with PLEO in the indicated concentrations for 8 h, followed determination of the cell proliferation by MTS assay. Data are mean ± SE of three independent experiments. The cell proliferation was normalized as percentage of drug-free control at the indicated doses. ^*^P<0.05 compared to the value before PLEO application. (B) YD-8 cells were treated without or with PLEO in the indicated concentrations for 8 h. The number of surviving YD-8 cells was counted under a microscope. Data are mean ± SE of three independent experiments. The number of surviving cells was normalized as percentage of drug-free control. ^*^P<0.05 compared to the value in the absence of PLEO. (C) YD-8 cells were treated without or with PLEO (60 μg/ml) for 8 h. Extra-nuclear fragmented DNA from the conditioned cells was then extracted and analyzed on a 1.7% agarose gel. The image is a representative of three independent experiments.

Initially, we measured the effect of PLEO in different concentrations on proliferation of YD-8 cells by MTS assay. As shown in Fig. 1A, while treatment with PLEO at 20 μg/ml for 8 h did not affect YD-8 cell proliferation, that with PLEO at 40 and 60 μg/ml inhibited the cell proliferation by 30 and 60%, respectively. Cell count analysis was next carried out to test the effect of PLEO on survival of YD-8 cells. As shown in Fig. 1B, treatment with PLEO at 20 or 40 μg/ml decreased survival of YD-8 cells by about 60%. PLEO treatment at 60 μg/ml further increased reduction of the cell survival by 70%. Due to strongest inhibitory effects on both proliferation and survival of YD-8 cells, we selected 60 μg/ml concentration of PLEO for further studies. Whether PLEO treatment induces apoptosis in YD-8 cells was next investigated by measuring nuclear DNA fragmentation, an apoptotic marker. As shown in Fig. 1C, compared with control (lane 1), there was strong induction of nuclear DNA fragmentation in YD-8 cells treated with PLEO at 60 μg/ml for 8 h (lane 2).

### Treatment with PLEO leads to activation of caspase-9, PARP cleavage and down-regulation of Bcl-2 in YD-8 cells

**Figure 2:**
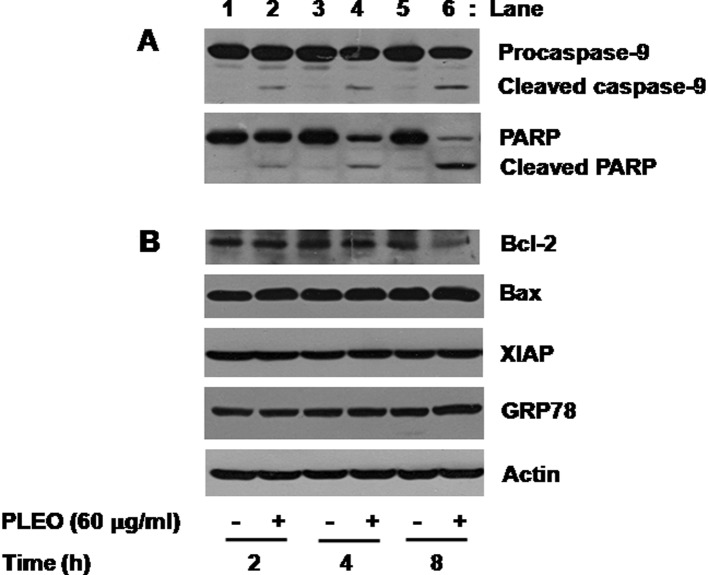
Effects of PLEO on expressions of caspase-9, PARP, Bcl-2, Bax, XIAP and GRP78 in YD-8 cells. (A, B) YD-8 cells were treated without or with PLEO (60 μg/ml) for the indicated times. At each time, whole cell lysates were prepared and analyzed by Western blotting. The image is a representative of three independent experiments.

We next investigated the molecular and cellular mechanisms and/or factors leading to the PLEO-induced apoptosis and growth inhibition in YD-8 cells. We primarily determined the effect of PLEO on activities of caspases, a family of proteases involved in apoptosis, in YD-8 cells using Western blot analysis. Activation of caspases, herein caspase-9, was assessed by measuring the amounts of inactive proform of caspase-9 (procaspase-9) and of cleaved form (active) of caspase-9 in YD-8 cells. As shown in Fig. 2A (panel 1), compared with control (lanes 1, 3 and 5), treatment of YD-8 cells with PLEO (60 μg/ml) at 2 h induced generation of cleaved caspase-9 (lane 2). The PLEO-induced generation of cleaved caspase-9 was further detected by the time of 4 or 8 h (lane 4 or 6). To confirm the PLEO-induced activation of the caspase pathway, we next determined the effect of PLEO on expression of PARP, a known substrate of caspases, in YD-8 cells. As shown in Fig. 2A (panel 2), treatment with PLEO resulted in a time-dependent generation of cleaved PARP (lanes 2, 4 and 6). Next, the effect of PLEO on expression of anti-apoptotic proteins, such as Bcl-2, Bax and XIAP, in YD-8 cells was investigated. As shown in Fig. 2B (panel 1), compared with control (lanes 1, 3 and 5), treatment with PLEO at 2 or 4 h had little effect on expression of Bcl-2 (lane 2 or 4), but PLEO treatment at 8 h strongly repressed Bcl-2 expression (lane 6). As shown in Fig. 2B (panels 2 and 3), however, expressions of Bax and XIAP were not changed in YD-8 cells treated without or with PLEO at the times tested (lanes 1–6). We also examined whether PLEO induces ER stress in YD-8 cells by measuring expression of GRP78, an ER stress-inducible protein. PLEO treatment did not modulate expression of GRP78 in YD-8 cells (Fig. 2B, panel 4, lanes 1–6). Expression of control actin protein was not affected in YD-8 cells treated without or with PLEO at the times tested (Fig. 2B, panel 5, lanes 1–6).

### Treatment with PLEO leads to activation of ERK-1/2 and JNK-1/2 in YD-8 cells

**Figure 3:**
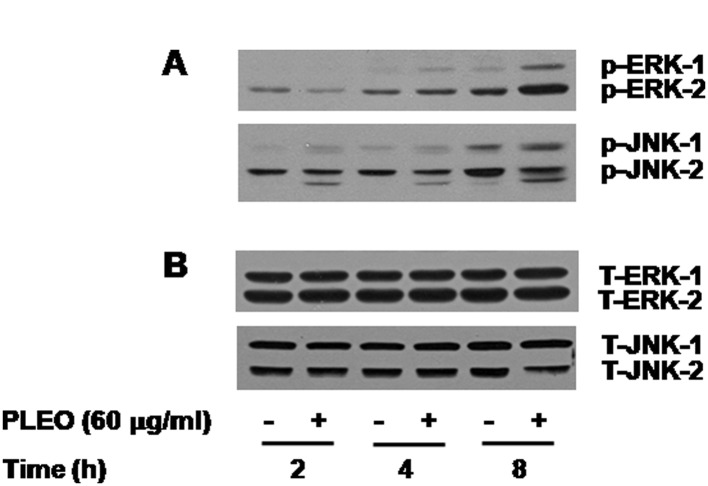
Effects of PLEO on phosphorylation of ERK-1/2 and JNK-1/2 in YD-8 cells. (A) YD-8 cells were treated without or with PLEO (60 μg/ml) for the indicated times. At each time, whole cell lysates were prepared and analyzed by Western blotting. The image is a representative of three independent experiments. (B) The same immunoblot as (A) was stripped and then reprobed with an antibody which recognizes total expression levels of ERK-1/2 or JNK-1/2. The image is a representative of three independent experiments. p-ERK-1/2, phosphorylated ERK-1/2; T-ERK-1/2, total ERK-1/2; p-JNK-1/2, phosphorylated JNK-1/2; T-JNK-1/2, total JNK-1/2.

We also determined the effect of PLEO on activities of the family of MAPK in YD-8 cells. In this study, activation of the MAPK family, including ERK-1/2, JNK-1/2 and p38 MAPK, was assessed by measuring phosphorylation level of each protein in YD-8 cells treated without or with PLEO (60 μg/ml). As shown in Fig. 3A (panel 1), compared with control (lane 1 or 3), there was little effect on phosphorylation level of ERK-1/2 by treatment with PLEO at 2 or 4 h (lane 2 or 4). However, PLEO treatment at 8 h largely increased phosphorylation level of ERK-1/2 in YD-8 cells (lane 6) compared with control (lane 5). Notably, as shown in Fig. 3B (panel 2), compared with control (lanes 1, 3 and 5), treatment with PLEO at 2 h slightly enhanced phosphorylation level of JNK-1/2 (lane 2) and the enhanced JNK-1/2 phosphorylation sustained by the time of 4 or 8 h (lane 4 or 6). However, there was no detection of phosphorylated forms of p38 MAPK in control YD-8 cells and PLEO treatment at the times tested did not modulate phosphorylation of p38 MAPK in YD-8 cells (data not shown). As shown in Fig. 3B, Western blot analysis applying an antibody which recognizes total expression levels of ERK-1/2 or JNK-1/2 into the stripped immunoblot used in Fig. 3A demonstrated no change of total expression levels of ERK-1/2 (lanes 1–6, panel 1) or JNK-1/2 (lanes 1–6, panel 2) in YD-8 cells treated without or with PLEO at the times tested, suggesting the ability of PLEO to increase phosphorylation levels of pre-existed ERK-1/2 and JNK-1/2 in YD-8 cells without *de novo* protein synthesis of these proteins.

### Treatment with PLEO leads to generation of intracellular ROS in YD-8 cells

**Figure 4:**
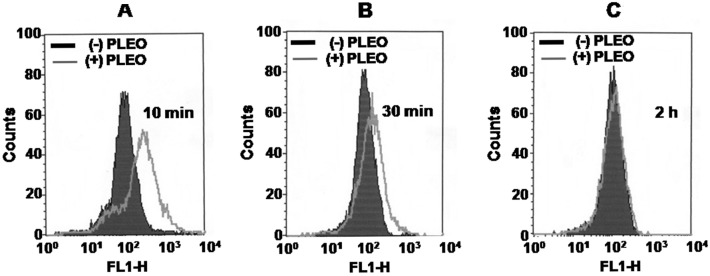
Effect of PLEO on generation of intracellular ROS in YD-8 cells. YD-8 cells were primarily loaded with DCFH-DA (20 μM) for 20 min and then treated without or with PLEO (60 μg/ml) for 10 min (A), 30 min (B) or 2 h (C). At each time, cells were harvested, washed twice with PBS and suspended in PBS. The ROS generation was measured by the DCF fluorescence intensity (FL-1, 530 nm) from 10,000 cells with a FACS Calibur flow cytometer (Becton-Dickinson). Data analysis was carried out using CellQuest program. The fluorescence is expressed as a histogram. Each picture in (A), (B) or (C) is a representative of three independent experiments.

Whether PLEO treatment alters intracellular levels of ROS in YD-8 cells was next determined by flow cytometry analysis. As shown in Fig. 4A and B, compared with control (dark line), treatment of YD-8 cells with PLEO at 10 min led to generation of intracellular ROS (grey line). The PLEO-induced ROS generation was further detected at the time of 30 min. However, there was no detection of intracellular ROS in YD-8 cells after treatment with PLEO at 2 h (Fig. 4C) and thereafter (data not shown).

### Generation of ROS and activities of caspases are important for the PLEO-induced growth inhibition and apoptosis in YD-8 cells

**Figure 5:**
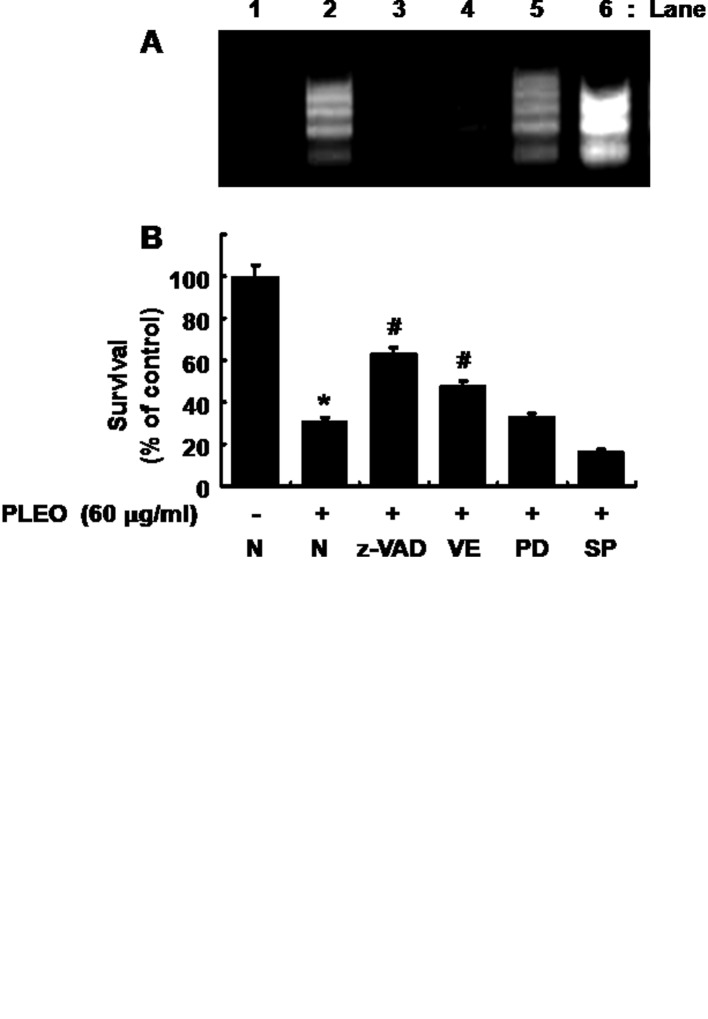
Effects of z-VAD-fmk, vitamin E, PD98059 or SP600125 on DNA fragmentation, reduction of survival, activation of caspase-9, PARP cleavage and/or Bcl-2 down-regulation induced by PLEO in YD-8 cells. (A, B) YD-8 cells were pretreated without or with a pan-caspase inhibitor z-VAD-fmk (z-VAD, 100 μM), an anti-oxidant vitamin E (VE, 100 μM), an ERK-1/2 inhibitor PD98059 (PD, 50 μM) or a JNK-1/2 inhibitor SP600125 (SP, 25 μM) for 1 h and treated without or with PLEO (60 μg/ml) for additional 8 h. (A) Nuclear DNA was then extracted from the conditioned cells and analyzed on a 1.7% agarose gel. The image is a representative of three independent experiments. (B) The number of surviving cells was counted under a microscope. Data are mean ± SE of three independent experiments. ^*^P<0.05 compared to the value of control (no PLEO); ^#^P<0.05 compared to the value of PLEO treatment in the absence of z-VAD, VE, PD or SP. (C) YD-8 cells were pretreated without or with z-VAD-fmk (z-VAD, 100 μM) or vitamin E (VE, 100 μM) for 1 h and treated without or with PLEO (60 μg/ml) for additional 8 h. Whole cell lysates were prepared and analyzed by Western blotting. The image is a representative of three independent experiments.

Whether ROS generation and/or activation of caspases, ERK-1/2 and JNK-1/2 is necessary for the PLEO-induced growth inhibition and/or apoptosis in YD-8 cells was next investigated using different pharmacological inhibitors, including z-VAD-fmk (z-VAD, a pan-caspase inhibitor), PD98059 (an inhibitor of ERK-1/2), SP600125 (an inhibitor of JNK-1/2) or vitamin E (an anti-oxidant). As shown in Fig. 5A, the PLEO-induced nuclear DNA fragmentation (apoptosis) (lane 2) was strongly inhibited by treatment with z-VAD-fmk (lane 3) or vitamin E (lane 4), but not with PD98059 (lane 5) or SP600125 (lane 6); rather the JNK-1/2 inhibitor enhanced the stimulating effect of PLEO on nuclear DNA fragmentation. Moreover, as shown in Fig. 5B, the PLEO-induced reduction of YD-8 cell survival (column 2) was effectively blocked by the caspase inhibitor (column 3) or the anti-oxidant (column 4), but not with the ERK-1/2 inhibitor (column 5) or the JNK-1/2 inhibitor (column 6).

### Evidence that ROS generation lies upstream of activation of caspases, PARP cleavage and Bcl-2 down-regulation in response to PLEO exposure

We further tested any crosstalk among ROS generation, activation of caspases and/or Bcl-2 down-regulation induced by PLEO in YD-8 cells. As shown in Fig. 5C (panels 1 and 2), as expected, the PLEO-induced activation of caspase-9 and PARP cleavage in YD-8 cells (lane 2) was suppressed by treatment with z-VAD-fmk (lane 3). However, as shown in Fig. 5C (panel 3), the PLEO-induced Bcl-2 down-regulation in YD-8 cells (lane 2) was not affected by z-VAD-fmk (lane 3). Of note, as shown in Fig. 5C (panels 1–3), the PLEO-induced activation of caspase-9, PARP cleavage and Bcl-2 down-regulation (lane 2) was effectively blocked by vitamin E (lane 4). Expression of control actin protein remained constant in YD-8 cells treated without or with PLEO in the absence or presence of z-VAD or vitamin E (Fig. 5C, panel 4, lanes 1–4).

### Treatment with PLEO also has strong anti-survival effect on other types of human OSCC cells

**Figure 6:**
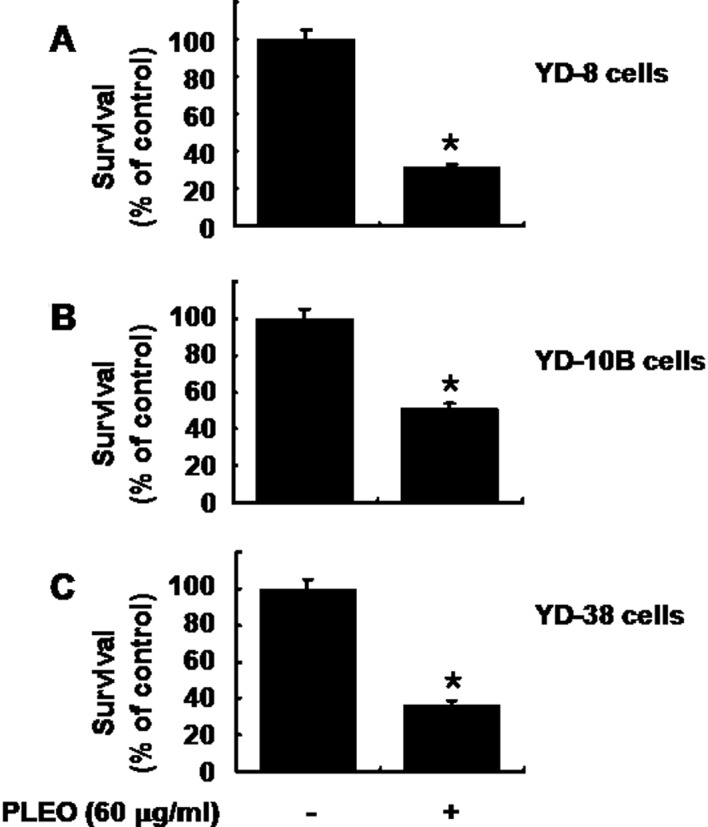
Effects of PLEO on survival of different human OSCC cell lines. Three different human OSCC cell lines, including YD-8 (A), YD-10B (B) and YD-38 (C), respectively, were treated without or with PLEO (60 μg/ml) for 8 h. The number of surviving cells, respectively, was counted under a microscope. Data are mean ± SE of three independent experiments. The number of surviving cells was normalized as percentage of drug-free control. ^*^P<0.05 compared to the value in the absence of PLEO.

We carried out additional cell culture experiments to see whether PLEO treatment affects growth of other human OSCC cell lines, including YD-10B and YD-38. As expected, treatment of YD-8 cells with PLEO (60 μg/ml, 8 h) decreased the cell survival by 68% (Fig. 6A). Treatment with PLEO (60 μg/ml, 8 h) also decreased survival of YD-10B and YD-38 cells by about 50 and 60%, respectively. These results suggest that the growth suppressive effect of PLEO is not limited to YD-8 cells.

## Discussion

In the present study, we investigated the anticancer activity of essential oil extracted from the leaf of *P. densiflora*, a pine tree widely distributed in Asian countries that has been used as a traditional medicine. Our data show that *P. densiflora* leaf essential oil (PLEO) has anti-proliferative, anti-survival and pro-apoptotic effects on YD-8 cells and the effects are associated with ROS generation, activation of caspases and Bcl-2 down-regulation.

In initial experiments, we showed that treatment with PLEO (60 μg/ml, 8 h) inhibits proliferation and survival of YD-8 cells (Fig. 1A and B). It is well understood that cells undergoing apoptosis have distinct biochemical and morphological characteristics, such as nuclear DNA fragmentation (4,23). Thus, considering that PLEO treatment leads to strong nuclear DNA fragmentation in YD-8 cells (Fig. 1C), it is obvious that PLEO treatment induces death of YD-8 cells by apoptosis.

Induction of apoptosis is largely influenced by a variety of cellular factors and proteins. Oxidative stress, which often occurs due to imbalance of intracellular levels of oxidants (e.g., ROS) and reducing agents (e.g., glutathione), is one of key cellular factors involved in induction of apoptosis. The role of ROS in apoptosis induction and/or cell death signaling has been reported (9,10). Interestingly, a recent study has shown that essential oil from *Zanthoxylium schinifolium*, an aromatic plant induces ROS-dependent apoptosis in human liver cancer cells (24). In this study, we have demonstrated that PLEO treatment even at 10 min is able to increase intracellular levels of ROS in YD-8 cells (Fig. 3), suggesting that PLEO treatment rapidly induces oxidative stress by ROS generation in YD-8 cells. Importantly, the present data with strong blockage of the PNEO-induced apoptosis in YD-8 cells and reduction of their survival by vitamin E, an anti-oxidant (Fig. 5A and B) further imply that ROS generation is critical for the PLEO-induced apoptosis and growth inhibition in YD-8 cells.

Previously, the potential role of caspases in certain plant-derived essential oil-induced apoptosis in human cancer cells has been suggested. For example, it is shown that caspases are involved in induction of apoptosis by essential oil of *Curcuma wenyujin*, a perennial herbal plant in human liver cancer cells (25). Of interest, it has been reported that activation of caspases is critical for apoptosis induced by essential oil isolated from *Artemisia iwayomogi*, a perennial herbal plant in human oral epidermoid cancer cells (26). In resting cells, caspases are expressed in a precursor form (inactive) with certain molecular weight. However, when cells are exposed to apoptogenic stimuli, they are processed via partial proteolytic cleavage and activated. Once activated, caspases cleave many cellular proteins, including PARP and other vital proteins, leading to induction and/or execution of apoptosis (27,28). Therefore, assuming that PLEO treatment induces activation of caspase-9 in YD-8 cells (Fig. 2) and treatment with z-VAD-fmk, a pan-caspase inhibitor inhibits the PLEO-induced DNA fragmentation in YD-8 cells and reduction of their survival (Fig. 5A and B), it is likely that activation of the caspase pathway is also important for the PLEO-induced apoptosis and growth suppression in YD-8 cells.

Substantial evidence suggests that the members of Bcl-2 family are also involved in apoptosis initiation and caspase activation by regulating the mitochondrial membrane integrity (6,29). It was previously shown that lower expression level of Bcl-2 is associated with mitochondrial dysfunction, resulting in the release of intermembrane proteins, such as cytochrome c, that function in the activation and assembly of caspases, such as caspase-9 (30). Evidence further indicates high expression level of Bcl-2 protein with poor survival in OSCC (31–33) and that low expression level of Bcl-2 correlates with high expression level of pro-apoptotic Bax protein, which would promote apoptosis of OSCC (32). In view of this, it is interesting to note recent studies that treatment with pine needle-derived essential oil induces apoptosis in human liver cancer cells and Bcl-2 expression is down-regulated in the essential oil-induced apoptotic cells (21) and the apoptotic response in human oral epidermoid cancer cells to essential oil from *Artemisia iwayomogi* is in part mediated via Bcl-2 down-regulation (26). In this study, we have shown that PLEO treatment only at 8 h decreases expression of Bcl-2 in YD-8 cells, but Bax expression is not affected by PLEO treatment at the times tested (Fig. 2B). The family of human IAP, including XIAP, is another suppressor of apoptosis (7) and inhibitor of caspases, including caspase-9 (34,35). In this study, we have demonstrated that PLEO treatment does not influence expression of XIAP in YD-8 cells (Fig. 2B). Given that activation of caspase-9 is inducible at 2 h PLEO treatment in YD-8 cells (Fig. 2A), it is obvious that early activation of the caspase-9 in the PLEO-treated YD-8 cells is not through modulation of Bcl-2 expression but via other mechanisms. Importantly, the present study provides experimental evidence that ROS generation lies upstream of activation of caspase-9 and Bcl-2 down-regulation in response to PLEO treatment, as demonstrated by ROS generated at 10 min PLEO treatment in YD-8 cells (Fig. 4A) and that PLEO-induced both activation of caspase-9 and Bcl-2 down-regulation is not shown by treatment with vitamin E, an anti-oxidant (Fig. 5C). Thus, it is likely that PLEO treatment rapidly increased intracellular ROS, which subsequently leads to activation of caspase-9 and down-regulation of Bcl-2 in YD-8 cells.

Evidence suggests that activities of the family of MAPK are also linked to cell proliferation, survival and/or apoptosis. It is previously shown that ERK-1/2 is phosphorylated and activated in cells upon exposure to mitogenic stimuli and the activated ERK-1/2 facilitates cell proliferation and/or transformation (36). On the other hand, JNK-1/2 and/or p38 MAPK are activated in cells exposed to stressful conditions (37) and often linked to induction of apoptosis (38). In a recent study, it has been demonstrated that treatment with essential oil from *Artemisia iwayomogi* leads to activations of ERK-1/2, JNK-1/2 and p38 MAPK and the activation is important for the mitochondrial- and caspase-dependent apoptotic death of human oral epidermoid cancer cells (26). The present study, however, demonstrates that though PLEO treatment leads to activation of ERK-1/2 and JNK-1/2 in YD-8 cells (Fig. 3), their activation is not necessary for the PLEO-induced apoptosis and growth inhibition in YD-8 cells, which is deduced from no effect of the PLEO-induced DNA fragmentation in YD-8 cells and reduction of their survival by treatment with PD98059 (an ERK-1/2 inhibitor) or SP600125 (a JNK-1/2 inhibitor) (Fig. 5A and B).

It is obvious that PLEO has anti-proliferative, anti-survival and pro-apoptotic effects on YD-8 cells. However, at present, it remains unclear whether the effects are mediated through whole extract of PLEO or some component(s) in it. Through GC and GC-MS analyses, in this study, we have indentified 17 compounds in PLEO, of which 2,2-dimethyl-3-methylenenorbornane (22.38%), α-pinene (20.58%), α-limonene (20.16%) and bornyl acetate (9.79%) are the main constituents (data not shown). Interestingly, studies have recently shown that essential oil *Tanacetum gracile* with high contents of α-pinene induces mitochondrial-dependent apoptosis in human leukemia cells (39) and treatment with α-pinene (150 μg/ml) isolated from *Schinus terebinthifolius Raddi* is able to induce apoptosis in a murine melanoma cell line (40). Considering these previous reports and the present observation of high contents of α-pinene in PLEO, it is conceivable that α-pinene may be one of the bioactive compounds in PLEO leading to the cytotoxic and/or apoptotic effect. However, in this study, we observed that single treatment with α-pinene has little effect on growth of YD-8 cells (data not shown). It could be informative to indentify which component(s) in PLEO, except α-pinene, exerts the cytotoxic and/or apoptotic effects on YD-8 cells.

Cancer cells and/or tissues may differ in many ways, such as in their cell of origin, the molecular alterations causing them and the susceptibility and defenses of the patient, and this makes the choice of appropriate treatment more difficult. Thus, drugs or agents which inhibit growth and/or induce apoptosis in different origins of cancer cells may be good anticancer strategies. In view of this, it is important to note the ability of PLEO to inhibit survival of not only YD-8 but also YD-10B and YD-38, other types of OSCC cells.

In conclusion, we demonstrated for the first time that essential oil extracted from the leaf of *P. densiflora* has strong anti-proliferative, anti-survival and pro-apoptotic effects on YD-8 cells and the effects are largely due to the ROS-dependent activation of caspases and Bcl-2 down-regulation. It is suggested that *P. densiflora* leaf essential oil has potential as an anticancer agent against human OSCC.
